# Exploring Parenting Styles Patterns and Children’s Socio-Emotional Skills

**DOI:** 10.3390/children10071126

**Published:** 2023-06-29

**Authors:** Aikaterini Vasiou, Wassilis Kassis, Anastasia Krasanaki, Dilan Aksoy, Céline Anne Favre, Spyridon Tantaros

**Affiliations:** 1 Department of Primary Education, University of Crete, 74100 Rethymno, Greece; 2 Department of Research & Development, School of Education, University of Applied Sciences and Arts Northwestern Switzerland, 5210 Windisch, Switzerland; wassilis.kassis@fhnw.ch (W.K.); dilan.aksoy@fhnw.ch (D.A.); celineanne.favre@fhnw.ch (C.A.F.); 3 School of Humanities, Hellenic Open University, 26335 Patras, Greece; akrasanaki@hotmail.com; 4 Department of Psychology, National and Kapodistrian University of Athens, 15784 Athens, Greece; sgtan@psych.uoa.gr

**Keywords:** parenting styles, SDQ, latent profile analysis, externalizing behavior problems, internalizing behavior problems, prosocial behavior, socio-emotional development

## Abstract

In this study, we adopted parenting styles as a multidimensional and latent construct that includes different aspects of parenting, rather than solely focusing on a single parenting style. In a Web-based survey with 1203 Greek parents, we identified parenting styles and their SDQ reports on their children. According to our results by Latent Profile Analysis, we must use a more complex approach concerning parenting styles. We identified a “Highly Authoritative style” profile with high levels of authoritative, low levels of authoritarian and middle levels of permissive parenting styles. We additionally identified a profile called “Relaxed Authoritative style”, with still high but lower levels of authoritative style, low but slightly heightened levels of authoritarian style, and middle levels of permissive style. A further profile, named “Permissive Focused Authoritative style”, had a mix of high levels of authoritative, moderate levels of permissive, and elevated levels of authoritarian parenting styles. Finally, in a profile named “Inconsistent Parenting style”, we identified parents with a blend of still high, but the lowest of all four levels of authoritative and highest levels of permissive and authoritarian parenting styles. When combining the four identified parenting patterns with the SDQ results, we identified the “highly authoritative parenting style” profile to be the least connected to internalizing or externalizing problems of the respective children.

## 1. Introduction

Bronfenbrenner’s socioecological framework [1], understanding development as a social process, as a result of the interaction between people and their environment, suggests that, among the proximal (microsystem) socialization agents, parents play the most pivotal role in children’s development [2]. As such, researchers have conducted numerous empirical investigations which focused on isolating the contribution of parenting styles to children’s and adolescents’ behavior, such as externalizing and internalizing problems and prosocial behavior [3,4,5]. Nevertheless, most studies have adopted variable-centric perspectives to explore the overall strength of associations between the examined variables in a larger population. Additionally, this approach primarily used single parenting styles, such as authoritarian, permissive, or authoritative, to understand the effects of particular parental behavior. This led to the understanding that parents apply a specific and characteristic style. Nevertheless, the use of analytical techniques, such as latent profile analysis, focused on people characteristics, often intervenes with the possibility of developing a fully realized understanding of the predictors and outcomes of within-subject variation in parenting styles [6,7,8]. Consequently, the present study is conducted to identify unique parenting style profiles and considers parenting styles as a multidimensional and latent construct that includes different aspects of parenting, not just a single one. Using latent profile analysis and exploring the possible relationship between the identified profiles and children’s and adolescents’ externalizing and internalizing problems and prosocial behavior, we attempt to develop a more complex and adequate picture of parenting styles and their effects on children’s behavior.

### 1.1. Parenting Styles

Parenting style is a collection of parents’ attitudes, behaviors, and emotions [9]. Therefore, we can conceptualize parenting styles as representing general types of child-rearing that characterize parents’ typical strategies and responses [10]. In particular, parental behavior is established in four specific behavioral dimensions: control, maturity demands, clarity of communication, and nurturance [11,12,13,14]. Baumrind [15,16], resulting from a blending of these dimensions and not from any single one of them, identified three primary parenting styles: Authoritarian (high levels of control and maturity demands, and low levels of nurturance and clarity of communication), Permissive (high levels of nurturance and clarity of communication, and low levels of control and maturity demand) and Authoritative (control, nurturance, clarity of communication, and maturity demands).

The behavior of authoritarian parents, which is not characterized by parental responsiveness and emotional availability, but by parental control, leads to limiting the independence of the children, valuing blind obedience, as well as adopting aggressive one-way communication [17,18,19]. In contrast to the authoritarian parenting style, permissive parents encourage responsiveness in the absence of parental control and maturity requirements as well as clear, consistent discipline and communication [17,19,20]. Permissive parenting style includes loose or contradictory discipline, indifference to the child’s disobedience, and lack of confidence regarding the parental role [18]. Permissive parents rarely punish, encourage independence, and offer unconditional support to their children [17,19]. On the other hand, authoritative parents emphasize responsiveness and control. They avoid, however, interacting with their children by being intrusive, criticizing, scolding, and threatening, as authoritarian parents are wont to do [17,21]. In addition, they try to understand the needs of their children. They behave warmly, lovingly, and dialogically. They also provide guidance and direction through suggestions, explanations, and argumentation. In addition, they set developmentally appropriate expectations while demonstrating receptivity and flexibility by offering children opportunities to practice independence and autonomy [17,22,23].

### 1.2. Children’s Behavior Problems

Empirically formulated classifications of child behavior have distinguished social-emotional and behavior problems as externalizing and internalizing [24]. Externalizing problem behaviors are considered aggressive and dysfunctional conducts aimed at others, while internalizing problem behaviors refer to negative emotions and moods such as depression, anxiety, and guilt [25,26,27]. Both aspects of dysfunction are related to impaired academic, social, and emotional development in children, such as the risk of several poor outcomes, including poor peer relationships, underachievement at school, poor personal adjustment, and poor mental health [28,29,30].

In detail, externalizing problems include the most common childhood disorder, attention deficit hyperactivity disorder (ADHD), in which a wide range of cognitive, interpersonal, social, emotional, and behavioral difficulties exist. Its main characteristics are aggression and disobedience, while other Disruptive Behavioral Disorders often coexist [31,32,33,34]. In addition, externalizing problems include conduct problems and behavioral disorders, in which the child comes into conflict with the environment, such as aggression, violence, reactivity, disobedience, drug use, and delinquency, problems which are observed when there is a lack of parental support [18,35,36,37]. Parental supervision, parental care, and normative parental expectations constitute the three dimensions of parenting that are systematically related to multiple behavioral problems in children and adolescents [38]. Research found associations between extreme types of control (harsh parenting, physical punishment, psychological control, overprotection, overactive parenting) or lack of control and supervision, and a strong presence of externalizing problems, such as conduct problems [33,39]. It is further considered that ineffective parenting (corporal punishment, inconsistency, poor supervision, and low involvement) and specific personal characteristics, such as sentimentality, also lead to externalizing problems [40]. Especially for girls, the lack of parental support is an essential factor in developing behavioral problems [41]. Moreover, the manifestation of externalizing problems leads to peer victimization and vice versa [42].

Conversely, problems related to depression, anxiety, shyness, irritability, withdrawal, low self-esteem, poor physical health, negative relationships with peers and emotional problems are recognized as internalizing problems [18,36,37,43], which possibly stem from early experiences of rejection [23]. Regarding peer relationships, research shows that children without close friends have internalizing problems, while peer rejection and victimization are associated with internalizing and externalizing problems [44]. The lack of parental supervision regarding their friends is associated with internalizing problems in girls. In contrast, the lack of parental supervision during free time is associated with internalizing problems for both sexes [43].

### 1.3. Children’s Prosocial Behavior

Prosocial behavior is the critical component in development that contributes to positive children’s and adolescents’ development and thriving [45]. Recent decades’ research has shown associations between prosocial behavior and social, emotional, and psychological benefits in children and adolescents, including better relationships with peers and adults [46,47,48,49,50], optimal functioning in terms of well-being [51], academic success [47,52,53,54,55,56], and positive mental health [57,58].

Prosocial behavior refers to all voluntary behaviors aimed to benefit others [57]. They are positive social acts that promote the well-being of others and self-motivated behaviors that benefit others, demonstrating the existence of social conscience [22,58,59,60]. Some manifestations of positive social behavior can be caring, comforting, sharing, cooperating, volunteering, donating, and offering physical or emotional help to others [58,59,60,61]. The demonstration of prosocial behavior by children and adolescents, however, is influenced by factors such as parents, peers, school, teachers, and culture [22,62].

### 1.4. Parenting Styles and Children’s Behavior

For many years, parenting style’s role in children’s and adolescents’ problem behavior was the main focus of research [63,64]. In particular, studies have examined the role of parenting styles in internalizing and externalizing problem behavior [65], both during childhood [66,67] and adolescence [68,69]. Prior studies also proposed that parenting styles are associated with adolescents’ prosocial behavior [70,71]. Specifically, warm and supportive parenting reinforces self-regulatory abilities, strengthens prosocial behavior and moral values, and enhances adolescents’ acceptance of others’ needs [72,73].

Regarding the influence of parental factors on children’s social behavior, it is considered that the mother–child relationship and parenting practices play an important role in children’s overall development and the development of externalizing and internalizing problems specifically, regardless of gender, during early adolescence [74,75]. Some research suggests that parental support may predict a reduction in adolescent anxiety and depression in addition to preventing future depressive behavior [76,77], whereas parental support also appears to influence the development of prosocial behavior in children and adolescents [78,79].

Regarding the authoritative parenting style, its high responsiveness and demandingness have been scientifically associated with fewer behavioral problems [36]. In particular, authoritative parents promote the development of social and academic skills during childhood and adolescence [80]. The authoritative parenting style also has associations with less disobedience, reduced tendency to internalize problems and dangerous behaviors, high ability to regulate behavior, increased self-perception for acceptance by peers, and better adjustment [23,36,37,38,80,81]. Moreover, the authoritative parenting style continues to have a positive effect on children’s development from childhood until later adulthood [36,79]. Finally, an authoritative parenting style helps children become more compassionate, helpful, and kind to other people [79].

Conversely, due to their high responsiveness and low demandingness, permissive parents do not seek control and authority over their children, guide them to regulate their behavior, and let them decide for themselves [36]. Thus, children of permissive parents learn to be passive and unresponsive in their interactions with others, developing antisocial behavior. As a result, they become dependent and present low levels of cognitive development and self-control, as well as low self-concept for acceptance by peers, especially in girls [36,80]. In addition, a permissive parenting style positively correlates with externalizing and internalizing behavioral problems and delinquency [36].

Concerning the authoritarian parenting style, which is characterized by low responsiveness and high demandingness, it is significantly positively correlated with reduced ability to regulate behavior, reduced self-esteem and self-confidence, low social skills, adjustment difficulties, depression, delinquency, externalizing and internalizing problems, aggression in boys and hyperactivity in girls, as well as problems in interactions with peers [23,36,80,81]. Thus, authoritarianism and corporal punishment, especially during childhood, can lead to suicide, depression in adulthood, or problems in the later stages of the child’s life [36]. Furthermore, regardless of the intramarital conflicts in the family, socioeconomic level, and children’s temperament, harsh punitive discipline predicts child aggression at school [82]. However, a ten-year longitudinal study [83] showed no relationship between early parental punitiveness and subsequent aggression. Furthermore, Moore and Eisenberg [84] found no negative relationship between authoritarian parenting style and children’s positive social development. Finally, according to research, psychologically controlling parenting has detrimental effects on the psychosocial development of adolescents, increasing the risk of externalizing and internalizing behavioral problems [85,86].

### 1.5. Recent Studies on Parenting Styles Using a Person-Centered Approach

The importance of exploring naturally occurring patterns of parenting styles is increasingly recognized by recent empirical studies. These studies use a person-centered approach, which permits researchers to include multiple parenting types and expand their understanding of the determinants and outcomes of parenting styles. For example, Carpenter and Mendez [87] examined longitudinal parenting profile differences in children’s behavioral adjustment by measuring aggression and hyperactive behavior of preschool children twice during the academic year. In their study, Bowers et al. [6], based on latent profiles of youth-reported parenting styles, examined the effect of parenting profiles in promoting positive youth development [6]. Kim et al. also used latent profile analyses in a three-wave longitudinal study lasting eight years, from early adolescence to emerging adulthood, to identify parenting profiles in Chinese American families and explore their consequences on adolescent adjustment [7], while Zhang et al. examined subtypes and their stability, and changes in Chinese maternal parenting style during early adolescence [88]. Grounded in Self-Determination Theory, multigroup latent profile analyses showed that the high monitoring–high autonomy support profile yielded the most optimal outcomes on adolescent adjustment, while the low monitoring–high psychological control profile yielded the worst [89]. More recently, Teuber et al. used longitudinal person-oriented perspectives to examine the stability and possible changes in autonomy-related parenting profiles and to further explore their consequences on adolescents’ academic and psychological functioning [8].

### 1.6. The Current Study

In the current study, to investigate children’s and adolescents’ behavior, we used the Strengths and Difficulties Questionnaire (SDQ) [90], which is globally the most widely used tool for assessing children’s social, emotional, and behavioral problems and has also been translated into over 60 languages [91]. The parent version of the scale has interestingly indicated good psychometric properties: validity evidence based on internal structure (i.e., internal consistency), test–retest reliability of the scores, and inter-rater agreement on the scores [92]. In addition, recent findings from nationally representative data from the United Kingdom (UK) demonstrated that parent SDQ ratings show measurement invariance across the broad developmental period from preschool to adolescence [93,94]. Supportive evidence for measurement invariance of the parent version of the SDQ have also been provided across informants [95,96], community and clinical samples [89], gender and age of the child [97] and parent education level [98]. Specifically, we chose the three-factor model, which makes a broader distinction between prosocial, internalizing, and externalizing behaviors and indicated the best-fitting model in a sample of parents with 4- to 17-years old children in the US [99]. Recent results [100] reveal that the parent-version of the SDQ was a suitable tool for use and comparison across different contexts during the COVID-19 pandemic.

In addition, to explore how the dimensions of control, maturity demands, clarity of communication, and nurturance are combined with different parenting profiles, we examined how specific parenting profiles are related to children’s and adolescents’ behavior problems and prosocial behavior, expanding previous research in this area [6,7,88]. A review of the existing literature indicates a lack of research that follows a person-centered approach to examine the effects of parenting styles on children’s and adolescents’ behavioral problems and prosocial behavior. Therefore, the current study was designed to address this gap by stiving to isolate unique parental profiles using latent profile analysis. We used the Parenting Styles and Dimensions Questionnaire (PSDQ) [101,102,103] which is known as one of the few psychometrically robust scales measuring parenting practices [104] and has also been used in multiple different cultural groups [105]. This choice is justified by the idea that parents can engage in practices that align with any parenting style at different moments to different degrees [106].

Based on prior research using person-centered analytic approaches, we hypothesize that we will identify distinct parental profiles using latent profile analysis (H1). Aiming to define the predictive utility of the identified parental profiles, we also hypothesize that types of parenting profiles will display statistically significant differences in children’s and adolescents’ externalizing/internalizing behavior problems and prosocial behavior (H2). Finally, attempting to isolate personal and demographic characteristics that predict parental profiles, we expected that demographic factors such as parents age [107,108] and education [109,110] would be associated with children’s and adolescents’ externalizing/internalizing behavior problems and prosocial behavior profile membership (H3) and expected that younger and more educated parents would be more authoritative and less authoritarian.

## 2. Materials and Methods

The study is a web-based survey conducted in Greece via the Internet, in the framework of the third author’s master thesis, which was approved by the Hellenic Open University. Participants were recruited to voluntarily fill in an electronic form questionnaire created on Google Forms and posted in parent groups on social media by the same author. To achieve a sufficient response rate [111], the questionnaire was distributed multiple times for a period of 1 month; November until December 2019. Participants were instructed through a debrief describing the objective of the study and the confidential nature of their participation. In addition, to remove potential biases, the form elaborated on issues of protection of privacy and ethics and provided contact details for the third author. Participants were asked to confirm that they had read the form and were willing to participate in the study. Upon receipt of this confirmation, they were directed to the measures described below. Participants were asked to respond to all the answers and informed that participation would be anonymous. Participation duration was 15 min.

One of the reasons for choosing this kind of survey is the nature of the characteristics of population support, as groups are frequently established in which personal experiences are shared [112]. This research method was selected to ensure a diverse group of participants with varying levels of education, social status, and age. Online surveys were chosen for their convenience in reaching potential respondents who may be spread out over a large geographic area [113].

1203 parents participated in the study; 90.9% were women, while 9.1% were men. A total of 54% were 41–50 years old, 34.8% were 31–40, 9% were 51–60, 2% were 20–30, and 0.2% were over 60 years old. Regarding marital status, 88% were married, 8% were divorced, 1.4% were cohabiting, 1.3% were unmarried, 0.9% were widowed, and 0.4% were separated. Regarding their educational level, 41.8% were University or Applied sciences graduates, 28.4% PhD or master’s degree holders, 16.9% were general or vocational high school graduates, 9.9% were vocational training graduates, 2% were high school or technical school graduates, and 1% students. In terms of their occupational status, 83.3% were employed, and 16.7% were unemployed. Regarding their children’s gender, 47.8% were female. Finally, in regard to their children’ s age, the children were 6–12 (61.3%) and 13–18 (38.7%). By the DETECTANOMALY-procedure in SPSS (IBM, 2021), an option for detecting anomalies, we identified two cases out of 1205, which had to be removed because of their high anomaly index (case 933 = 9.70, respectively, for case 987 = 13.20) regarding the three parenting styles. Due to this, the analyses were performed with N = 1203 participants.

## 3. Measures

### 3.1. Parenting Styles

The Greek version of the Parenting Styles and Dimensions Questionnaire (PSDQ) by Robinson, Mandleco, Olsen, and Hart [101,102,103] was used, adapted to the Greek population by Maridaki-Kassotaki [102]. It is a self-administered questionnaire, grounded in Baumrind’s model of parental types based on two dimensions of parental behavior: responsiveness and demandingness [103]. It explores the parent–child relationship, communication, and parenting methods, distinguishing parents into three dominant parental types: the authoritative, the authoritarian, and the permissive types [102]. The sub-scale “authoritative parenting style” includes 12 statements (e.g., I understand my child’s feelings), the sub-scale “authoritarian parenting style” includes four statements (e.g., I use punishment as a means of discipline), and the sub-scale “permissive parenting style” includes three statements (e.g., I think it is hard to teach my child to discipline). All parenting scales had the same response format: 1 = never, 2 = sometimes, 3 = often, and 4 = always. Finally, regarding the internal consistency of the three parenting scales, Cronbach’s α was good for the authoritative type at 0.82, for the permissive type 0.75, and just satisfactory for the authoritarian type at 0.68.

### 3.2. Children’s and Adolescents’ Behavior

The parents’ version of the Strengths and Difficulties Questionnaire (SDQ) [90] was completed by participants, created to assess children’s and adolescents’ behavioral and emotional problems in their everyday life. Specifically, the Greek version of the Strengths and Difficulties Questionnaire—SDQ [90] was used and completed by parents of children and adolescents. The questionnaire has been adapted to the Greek population by Bibou-Nakou et al. [114]. It includes 25 statements and three answers (not true, somewhat true, and true). The main scale (of 25 statements) is made up of five sub-scales with five items each: 1. Hyperactivity/attention deficit (e.g., (S)He is restless and hyperactive, cannot remain calm, still for long periods of time), 2. Conduct disorder (e.g., (S)He often has tantrums or is irritable), 3. Relationships with peers (e.g., (S)He is rather lonely, tends to play alone), 4. Emotional disorders (e.g., (S)He often complains of headaches, stomach aches, or feeling sick) and 5. Positive social behavior with five items (e.g., (S)He takes into account the feelings of others). The subscales “Hyperactivity/attention deficit” and “Conduct disorder” make up the SDQ-dimension “Externalizing problems”. In contrast, the subscales “Relationships with peers” and “Emotional disorders” make up the SDQ-dimension “Internalizing problems”. The positive social behavior scale makes the SDQ-dimension “prosocial behavior”. As for internal consistency, Cronbach’s α was suitable for all three SDQ-dimensions: for the positive social behavior, 0.70; for externalizing problems, 0.76 and for internalizing problems, 0.71.

We performed the multinomial computations of banding scores, enabling us to identify non-clinical or “at risk/clinical” cases. To achieve this, we followed the same criteria employed by Goodman in the original version of the SDQ [90], supported by empirical research on the detection and prevalence of mental health issues [24,115]. Based on the fact that approximately 10% of children and adolescents exhibit some form of mental health problem, and another 10% have a borderline problem, we designated threshold values as follows: scores above the 80th percentile fall into the “at risk/clinical = 1” range, scores, and scores below the 80th percentile fall into the “non-clinical = 0” category [90,116,117]. This categorization was applied to all subscales except for Prosocial behavior, where scores equal to or below the 20th percentile was considered “at risk/clinical = 1” and scores below the 20th percentile were considered “non-clinical = 0”.

### 3.3. Covariates

Parents Education: To assess parents’ education level, we asked for the following six educational levels: University or Applied sciences graduates, PhD, or master’s degree holders, general or vocational high school graduates, vocational training graduates, high school or technical school graduates, and students.

Parents Age: Parents’ age was assessed by five categories: 20–30 years, 31–40 years, 41–50 years, 51–60 years, and over 60 years old.

## 4. Results

### 4.1. Analytic Strategy

The statistical analysis for this study was conducted in four steps: in step one, sociodemographic differences in the applied measures were examined using *t*-tests. In step two, because we regrouped the items to the scales, we performed a confirmatory factor analysis to test construct validity. In step three, parents’ parenting style patterns were identified by computing latent profile analyses (LPA) using three classification variables. In step four, we ran a multinomial regression analysis of the identified parenting style patterns related to SDQ to understand children and adolescents’ social behavior. For the conducted confirmatory factor analysis and the LPA, we used Mplus version 8.9 [118]. For the *t*-test and multinomial regression, SPSS 28 was used.

#### 4.1.1. Results Analytic Step One: Sociodemographic Differences of All Measures and Intercorrelations

We ran *t*-tests (see Table 1) to analyze for mean differences in the SDQ dimensions and parenting styles by age group of the respective children and adolescents. Referring first to the three introduced SDQ dimensions, we identified only small but still significant effects (displayed Cohen’s d is low) between children and adolescents, with children having higher externalizing problems. When comparing the levels of the three parenting styles, we identified significantly higher levels for younger children than older children for both authoritarian and permissive parenting styles.

When looking at the connections between the SDQ dimensions and the parenting styles that were found (as shown in Table 2), there were low to moderate intercorrelations, which means there was no issue with multicollinearity.

#### 4.1.2. Results Analytic Step Two: Confirmatory Factor Analysis (CFA) for Testing Construct Validity

To test for construct validity and to verify the factor structure we performed a confirmatory factor analysis. CFA allows testing of the assumption that a hypothesized relationship between observed variables and their underlying latent constructs exists. The RMSEA, TLI, and CFI are deemed particularly important for accurately estimating CFAs [119]. Following Marsh et al. [120], we established the benchmark for a satisfactory model fit as RMSEA values below 0.08, coupled with CFI and TLI values above 0.90 and SRMR values below 0.08, indicating a strong fit for the model. The fit indices obtained from the confirmatory factor analysis applied were sufficient for the three parenting style scales, as evidenced by the following: (χ^2^ (149) = 453.384, *p* < 0.001; RMSEA = 0.041 [90% CI = 0.037–0.046]; SRMR = 0.028 CFI = 0.923; TLI = 0.912), as for the five SDQ-parents sub-scales (χ^2^ (231) = 587.411, *p* < 0.001; RMSEA = 0.036 [90% CI = 0.032–0.039]; SRMR = 0.042 CFI = 0.926; TLI = 0.903). This confirms the construct validity for each scale of the study.

#### 4.1.3. Analysis Step Three: Identifying Parenting Style Patterns by Latent Profile Analysis (LPA)

We utilized three indicators, namely authoritarian, permissive, and authoritative parenting style, to group parents into distinct parenting style classes through the statistical application of Latent Profile Analysis (LPA). This allowed us to examine patterns of latent parenting styles, which encompassed multiple indicators and their interrelationships within the parenting style classes. By employing LPA as a comprehensive method, our objective was to assess the continuity of parenting style levels. The primary goal of this study was to use LPA to examine the proposed conceptualization of parenting styles, considering three aspects of parenting within an overarching latent structure, and to empirically classify latent variables into subgroups based on similar observations.

The models used in this study were non-nested. To determine the best model, different criteria were applied [121], including the entropy value, as well as information criteria such as the Akaike information criterion (AIC), Bayesian information criterion (BIC), and Sample-Adjusted BIC (ABIC). The smaller values indicate a better fit [122]. Entropy was also considered, with values above 0.7 deemed sufficient to indicate certainty in the estimation, but with models of entropy of 1.0 being overidentified [123,124]. The final latent profile analysis (LPA) model was chosen based on various statistical indicators and theoretical considerations. Additionally, model fit criteria such as the Vuong-Lo-Mendell-Rubin Likelihood Ration test (LMR-LRT), the Lo-Mendell-Rubin Adjusted Likelihood Ratio test (aLMR-LRT), and the Bootstrapped Likelihood Ratio test (BLRT) were used for the LPA. A significant *p*-value indicated an improvement to the previous model with k − 1 profiles. The ultimate model for an LPA, which determines the number of profiles, is selected based on a combination of statistical measures and pre-existing theoretical frameworks and the rule of the most parsimonious solution [125], which means that the interpretability and the additional information provided by a more complex solution has to be established. There are currently no established guidelines for determining the appropriate size of profiles [121]. Following Nylund [124], we are arguing against having profile sizes with less than 50 cases or these profiles being less than 5% of the total sample. 

The analysis was conducted for a range of two to six latent patterns. Statistical tests of model fit can be found in Table 3. A model consisting of four profiles was selected, as it had a lower aBIC score than a profile 3 solution, and the entropy was higher. For the comparison between the profile 3 the profile 4 solutions, we additionally applied model fit criteria with significant *p*-values for profile 3 over the profile two solutions, indicating an improvement to the previous model, but non-significant *p*-values on LMR-LRT and aLMR-LRT when comparing profile 3 and profile 4 but with still significant *p*-values on the BLRT, indicating an improvement for the profile 4 to the profile three models. When comparing the profile 4 to the 5 or 6 profile solution, we noticed several criteria decreasing. In comparison to the profile 4 solutions, we detected for profile 5 (aBIC Delta to profile 4 = 777) and 6 (aBIC Delta to profile 5 = 948) solutions a significant drop in aBIC differences, and for both solutions an Entropy of 1.0, which suggested weak evidence [126] and an overidentification of the model [127], leading us favoring the profile 4 solution.

For the 5 (one profile with *n* = 18 participants, 2.3% of the sample) and 6 profile (one profile with *n* = 49, 3.9% of the sample; one profile with *n* = 44, 3.6% of the sample; one profile with *n* = 9, 0.7% of the sample), solutions had far too small sample sizes [124,128]. Additionally, for both the 5 and 6 profile solutions, the new profiles did not offer new theoretical insights. but merely split already existing small profiles. Based on the abovementioned criteria and the principle of favoring more restricted and simple models, the profile 4 solutions were ultimately chosen. Along with empirical measures, the selection of the profile 4 solutions was also influenced by its interpretability and alignment with existing theoretical frameworks. 

**Figure 1:**
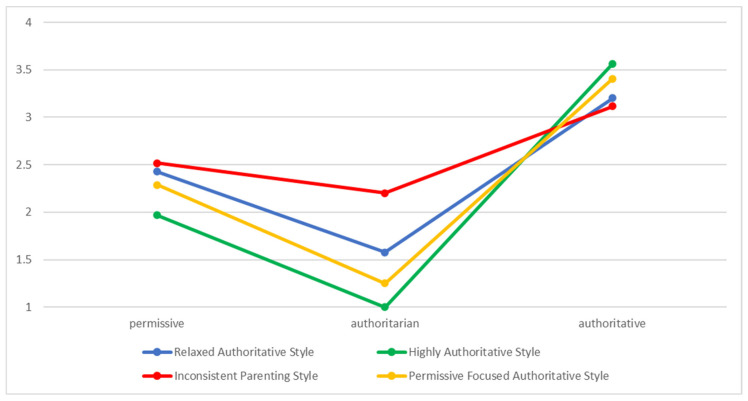
Diagrammatic Representation of the four identified parenting style profiles by LPA.

By the three introduced parenting styles and the consecutive tests on a different number of profiles (two to six profiles), we identified the four-profile solution as the best fitting. Regarding the distribution of the four profiles (see Figure 1), we identified a profile (profile 1, 66.6% of the participants) called Highly Authoritative style (HA) with high levels of authoritative, the lowest levels of authoritarian and middle levels of permissive parenting styles. We additionally identified a profile called Relaxed Authoritative style (RA) (profile 2, 16.3% of the participants) with still high but lower levels of authoritative style than in profile 1, low but elevated levels of authoritarian style, and middle levels of permissive style. Profile 4 (12.4% of the participants), named Permissive Focused Authoritative style (PFA), had a mix of the second highest levels of authoritative and middle levels of permissive and slightly higher levels of authoritarian parenting styles. Finally, in profile 3 (4.4% of the participants), named Inconsistent Parenting style (IP), we identified parents with a blend of higher levels of authoritative and middle levels of permissive and authoritarian parenting levels. From the solution chosen, we could detect that parenting styles are a complex mix and multidimensional latent construct encompassing authoritative, authoritarian, and permissive styles, rather than a distinct single parenting style as commonly assumed.

We analyzed if there were differences in the patterns concerning parental education and parents’ age to control for any effects caused by these two covariates by using multinomial-regression analysis. Neither for education (Wald chi2(12) = 9.830, *p* = 0.631) nor for age (Wald chi2(6) = 6.091, *p* = 0.413) have significant effects been identified.

#### 4.1.4. Analysis Step Four: Multinomial Regression Analysis on the Identified Parenting Patterns Related to the Three SDQ Dimensions to Understand the Social Behavior of the Respective Children

For the three SDQ dimensions (internalizing problems, externalizing problems, and prosocial behavior), we identified significantly lower levels of problems when comparing the “highly authoritative style” profile to the other three parenting profiles (see Table 4). This was especially the case when comparing the “highly authoritative style” profile to the “permissive focused authoritative style” or the “inconsistent parenting style” profile. No significant differences were identified (see Table 4) when comparing the levels of prosocial behavior of the four parenting profiles. In summary, we identified the “highly authoritative parenting style” profile to be the least connected to internalizing or externalizing problems of the respective children when studying the answers by their parents.

## 5. Discussion

Given the lack of studies that capture parenting styles as a heterogeneous construct and therefore solely focus on the individual and the well-known parenting styles, i.e., authoritative, authoritarian, and permissive, we pursued the research question as to whether there are distinct parenting style profiles. We conceptualized parenting style as a multidimensional and latent construct encompassing diverse aspects of parenting rather than a single one. We, therefore, defined parenting style as a collection, a mix of parents’ attitudes, behaviors, and emotions [9].

By using latent profile analysis and examining the association between the identified profiles and adolescents’ externalizing and internalizing behavior problems and prosocial behavior, our study is able to confirm the relevance of presenting the different parenting dimensions in a more complex and appropriate picture of parenting profiles and their influence on adolescents’ socio-emotional skills. Person-centered approaches extend beyond commonly used methods for establishing these parenting styles or profiles, such as the scale-mean or median-split methods, which can be problematic when dealing with multiple dimensions [129].

The present study adopted a person-oriented method to overcome these limitations and address the complex interplay of multiple dimensions. This approach allowed, following Hypotheses 1, identification of distinct parental profiles using latent profile analysis, for an adequate representation of the combinations of parenting styles. Interestingly, previous studies using person-centered approaches have revealed different combinations of parenting styles but have not confirmed distinct forms of permissive parenting profiles [88,110] or authoritarian profiles [130,131]. Our results supported these findings by considering parenting styles as a multidimensional construct rather than mere forms of distinct parenting styles. These findings build on previous research and demonstrate how person-oriented methods can provide insights that are difficult to achieve with variable-oriented techniques. Detecting the latent profiles used in this study to identify parenting styles would be challenging, if not impossible, to confirm using traditional variable-oriented analyses.

Confirming Hypothesis 1, we found four distinct profiles regarding a mix of all three parenting styles. We could not identify a parenting style that was uniquely focused on authoritarian, authoritative, or permissive styles, demonstrating that parenting styles should be captured as a multidimensional, latent concept. Interestingly, all four patterns were very high in the authoritative style, suggesting that some form of responsiveness and control characterizes all profiles. This finding is in line with other studies [87], which also found several parenting profiles consisted of authoritative (i.e., adaptive) parenting practices. Additionally, in our research, most parents had middle levels of authoritarian style (i.e., negative features). Specifically, three out of four profiles showed some authoritarian parenting style combined with authoritative and permissive styles. This means that a third of the children and adolescents do experience intrusive, critical, scolding, and threatening behaviors common to authoritarian parents [17,22,23], in addition to some levels of warm, loving, and dialogical behaviors [17,21], as well as loose or contradictory discipline [18].

Notably, analyses of the latent profile frequencies indicated that most parents in our sample perceived their practices as exhibiting a relatively positive parenting style/profile. Given that the concept of equifinality (i.e., different early experiences in life) is helpful for interpreting how parenting styles are associated with adaptive or maladaptive behavioral outcomes over time [87], the results of the current study extend the research on multiple manifestations of adaptive parenting by Greek parents of children and adolescents. We found it surprising that the permissive style was present to a moderate degree in all four profiles. This means that, although permissiveness alone is negative for socio-emotional development in children and adolescents, our results demonstrate that it was not determinant for profile affiliation in combination with high authoritative and low authoritarian styles.

Confirming Hypothesis 2, the present study demonstrated that the socio-emotional development in childhood and adolescence is strongly linked to the parenting style experienced. Children and adolescents with parents with primarily authoritative parenting styles, characterized by high levels of behavioral control and support and lower levels of psychological control, show a positive developmental status. In contrast, adolescents with affective controlling parents manifest problems in externalizing and internalizing behavior. This aligns with the existing empirical evidence, which consistently shows that the authoritative parenting style is positive for adaptive socio-emotional development, while the others are not [132,133,134]. These findings propose that children and adolescents have fewer behavioral problems [36] and a reduced tendency to internalize problems and dangerous behaviors [80,81].

Although the majority of parenting programs aimed at parents have focused on improving communication with their children, there are limited studies addressing parenting strategies [6,7,8]. Thus, we assume that parents may need more support in coping with their children’s behavioral problems and improving their parenting abilities to decrease the problem behavior. By identifying different patterns of parenting styles, it becomes clear that not all parents have the same needs. Interventions can be tailored to parents’ individual needs and challenges based on their specific profile patterns. This is important because, if parents can learn to create a positive and supportive environment for their children, they can reduce the risk of externalizing and internalizing behavioral problems, especially as parent–child conflict starts early in a child’s life and is very stable over time [31]. Thus, we adopt Teuber’s et al. [8] suggestion that the person-oriented results pointed out that it is useful to reinforce parents with guidance on positive parenting skills through parenting programs that focus on adaptive parenting practices, and direct the several maladaptive effects of different forms of dysfunctional practices. Contrary to our expectations regarding prosocial behavior, no significant differences were identified when comparing the levels of prosocial behavior of the four parenting profiles, supposing that our findings are inconsistent with prior findings that indicated that parenting dimensions are related to adolescents’ prosocial behavior [70,71]. Considering that we used SDQ parent reports regarding their children’s prosocial behavior, our study examined prosocial behavior as a global construct, ignoring differentiation between the subtypes of this behavior (e.g., altruistic, compliant, emotional, and public) [57], as well as between the motivations underlying it.

While our research on Hypotheses 3 challenges the assumption that parents’ age and education are strong determinants of parenting patterns [108,109,110], it is essential to note that the existing literature suggests some weak associations. Therefore, it is crucial to interpret our findings with caution. Nonetheless, our study underscores the need for further investigation into the multifaceted factors that influence parenting behaviors and the potential role of intervention programs, such as the newly developed profiles, in shaping these behaviors.

The implications of our findings on Hypothesis 3 are twofold. Firstly, it is suggested that other factors not considered in our research may have a more substantial impact on parenting patterns. It is possible that aspects such as cultural influences, personal values, or individual experiences may play a more significant role in shaping how parents interact with their children. Secondly, the reduced effects of parents’ age and education observed in our study could be attributed to the effectiveness of the newly developed profiles. These profiles might have facilitated a greater homogenization of parenting practices, potentially minimizing the impact of individual characteristics, such as age and education.

## 6. Limitations

Even if the insights gained by the chosen analytic design clearly expand the previous knowledge on parenting styles, there are a few limitations. As patterns of parental styles are not traits but states, we needed, instead of the chosen cross-sectional approach, a full longitudinal design. In future research, a latent transition analysis (LTA) should be applied to indicate significant differences in the longitudinal classification of the identified parenting patterns. LTA, the longitudinal extension of LCA, is a statistical tool that models possible parenting style pattern transitions over time. Especially. the findings regarding the “highly authoritative parenting style” as the least connected to children’s internalizing or externalizing problems should be approached with caution. There may be other confounding factors not considered in the analysis that could influence these associations, such as autonomy support and controlling parenting [135,136] or child–parent communication [137]. We also used parents’ self-perceptions of their parenting styles. Including the children’s perceptions of the respective parenting styles would have been interesting. Given that relations with parents play a distinct role in children’s development, the respective qualities of the relationship between parents and children are significant predictors of children’s academic, personal, and social development [138].

In addition, as our sample only included participants from a specific cultural context (Greek parents), the generalization of the findings to other countries and cultural contexts is rather limited. Furthermore, the sample restrictions and our specific sampling approach via the Internet can be considered another study limitation, even if our sample was large enough to be considered stable against minor deviations. Nevertheless, Mann and Stewart [139] noticed the risk of losing sight of who responds to online questionnaires. For example, about 90% of mothers answered our questionnaire. Although these surveys do not represent the total population of internet users, non-probability samples can be valuable, as they may be representative of a subgroup of the total population [113]. Another limitation is that marital status did not indicate if the parents were single mothers or fathers. In a future study, we could ask for this additional information, because it may matter to the chosen parenting styles [5]. We also did not ask for family income or migration status, both conditions that can also affect parenting styles [91,140].

## 7. Conclusions

To sum up, our results succeeded in extending parental types beyond the traditional authoritarian, permissive, and authoritative styles. The current study brings to light the person-centered approach in which parenting styles are better expanded into four parenting profiles, with the authoritative style predominating. Given the importance of the finding that one-third of children and adolescents exhibit behavior problems, the socio-emotional development in childhood and adolescence reaffirms the necessity of parenting programs to guide parenting practices.

## Figures and Tables

**Table 1:** Sample Mean Levels (and Standard Deviations) of the SDQ-Dimensions and Parenting Styles by Age Group of the Respective Child.

Variables	Range	6–12 Years Old (*n* = 431) M (SD)	>12–18 Years Old (*n* = 364) M (SD)	Cohen’s d
Internalizing problems (SDQ)	0–15	3.02 (2.85)	3.06 (2.67)	-
Externalizing problems (SDQ)	0–18	4.89 (3.26)	4.35 (2.99) **	0.17
Prosocial behavior (SDQ)	0–10	8.09 (1.81)	8.17 (1.74)	-
Authoritarian parenting style	1–4	1.19 (0.33)	1.13 (0.25) ***	0.20
Permissive parenting style	1–4	2.13 (0.62)	2.06 (0.62) *	0.12
Authoritative parenting style	1–4	3.46 (0.37)	3.49 (0.34)	-

Note. * = *p* < 0.05, ** = *p* < 0.01, *** = *p* < 0.001. between younger and older children.

**Table 2:** Intercorrelations of the SDQ-Dimensions and Parenting Styles.

Correlations						
	Internalizing Problems (SDQ)	Externalizing Problems (SDQ)	Prosocial Behavior (SDQ)	Authoritarian Parenting Style	Permissive Parenting Style	Authoritative Parenting Style
Internalizing problems (SDQ)	-					
Externalizing problems (SDQ)	0.41 ***	-				
Prosocial behavior (SDQ)	−0.24 ***	−0.35 ***	-			
Authoritarian parenting style	0.22 ***	0.31 ***	−0.16 ***	-		
Permissive parenting style	0.18 ***	0.31 ***	−0.15 ***	0.28 ***	-	
Authoritative parenting style	−0.16 ***	−0.30 ***	0.35 ***	−0.38 ***	−0.19 ***	-

Note. *** = *p* < 0.001.

**Table 3:** Model Fit Indices for Latent Profile Analysis on Parenting Styles, N = 1203.

	AIC	BIC	ABIC	Entropy	LMR LR Test *p*-Values	ALMR LR Test *p*-Value	Sample Proportion Per Profile (*n*; %)	Classification Accuracy	Blt *p*-Value
2-Profiles	3012	3063	3032	0.933	>0.05	>0.05	(138; 11.4%) (1065; 88.5%)	>0.921	<0.001
3-Profiles	2494	2566	2521	0.979	<0.001	<0.001	(949; 83.0) (151; 12.5) (53; 4.4)	>0.993	<0.001
4-Profiles	2174	2265	2208	0.995	>0.05	>0.05	(803; 66.7) (197; 16.3) (53; 4.4) (150; 12.4)	>0.996	<0.01
5-Profiles	1397	1509	1439	1.00	>0.05	>0.05	(197; 16.3) (103; 8.5) (71; 5.9) (803; 66.7) (18; 2.3)	1.00	<0.001
6-Profiles	449	581	498	1.00	>0.05	>0.05	(49; 3.9) (197; 16.3) (103; 8.5) (803; 66.7) (44; 3.6) (9; 0.7)	1.00	<0.001

Note. AIC = Akaike information criterion; BIC = Bayesian information criterion; ABIC = Sample-size adjusted BIC; LMR LR = Vuong-Lo-Mendell-Rubin Likelihood Ratio Test; ALMR LR = Lo-Mendell-Rubin Adjusted LRT Test; BLRT = Bootstrap likelihood ratio test.

**Table 4:** Multinomial logistic regression of SDQ-dimensions in the four LPA profiles.

LCA Wave 2 Profile	Predictor	B	SE	Wald Statistic	*p*	OR	Prediction in % Pseudo-R^2^		
							Cox & Snell	Nagelkerke	Mac-Fadden
Permissive focused authoritative style	Intercept	−0.88	0.18	23.73	<0.001		3.1	3.7	1.6
	SDQ-Internalizing	−0.99	0.21	22.62	<0.001	0.37			
Inconsistent Parenting style	Intercept	−1.60	0.24	44.72	<0.001				
	SDQ-Internalizing	−1.48	0.30	24.49	<0.001	0.23			
Relaxed Authoritative style	Intercept	−1.03	0.19	29.15	<0.001				
	SDQ-Internalizing	−0.44	0.21	4.39	<0.01	0.64			
Permissive focused authoritative style	Intercept	−0.74	0.18	17.15	<0.001		6.1	7.2	3.3
	SDQ-Externalizing	−1.19	0.21	33.16	<0.001	0.31			
Inconsistent Parenting style	Intercept	−1.18	0.21	32.19	<0.001				
	SDQ-Externalizing	−2.24	0.30	56.68	<0.001	0.11			
Relaxed Authoritative style	Intercept	−0.87	0.19	21.95	<0.001				
	SDQ-Externalizing	−0.64	0.21	9.56	<0.01	0.53			
Permissive focused authoritative style	Intercept	−1.13	0.18	39.62	<0.001		1.1	1.3	0.6
	SDQ-Prosocial behavior	−0.69	0.21	11.23	<0.001	0.50			
Inconsistent Parenting style	Intercept	−2.28	0.29	61.26	<0.001				
	SDQ-Prosocial behavior	−0.55	0.33	2.70	>0.05	0.58			
Relaxed Authoritative style	Intercept	−1.11	0.18	38.64	<0.001				
	SDQ-Prosocial behavior	−0.37	0.20	3.39	>0.05	0.69			

Note: S.E. = Standard Error; OR = Odds Ratio. Reference LPA profile is the profile we called “Highly Authoritative style”. For all three SDQ-dimensions: (0 normal; 1 at risk/clinical).

## Data Availability

The raw data supporting the conclusions of this article will be made available by the authors, without undue reservation.
